# Lipid ratios and appropriate cut off values for prediction of diabetes: a cohort of Iranian men and women

**DOI:** 10.1186/1476-511X-9-85

**Published:** 2010-08-17

**Authors:** Farzad Hadaegh, Masumeh Hatami, Maryam Tohidi, Parvin Sarbakhsh, Navid Saadat, Feridoun Azizi

**Affiliations:** 1 Prevention of Metabolic Disorders Research Center, Research Institute for Endocrine Sciences, Shahid Beheshti University of Medical Sciences, Yaman street, Velenjak, Tehran, Iran; 2 Endocrine Research Center, Research Institute for Endocrine Sciences, Shahid Beheshti University of Medical Sciences, Tehran, Iran

## Abstract

**Background:**

Dyslipidemia is a risk factor for incident type 2 diabetes; however, no study has specifically assessed the lipid ratios (i.e. total cholesterol (TC)/high density lipoprotein cholesterol (HDL-C) and triglyceride (TG)/HDL-C) as predictors of diabetes. We aimed to compare the independent association between the different lipid measures with incident diabetes over a median follow up of 6.4 years in Iranian men and women.

**Method:**

The study population consisted of 5201 non diabetic (men = 2173, women = 3028) subjects, aged ≥20 years. The risk factor adjusted odds ratios (ORs) for diabetes were calculated for every 1 standard deviation (SD) change in TC, log-transformed TG, HDL-C, non-HDL-C, TC/HDL-C and log-transformed TG/HDL-C using multivariate logistic regression analysis. Receiver operator characteristic (ROC) curve analysis was used to define the points of the maximum sum of sensitivity and specificity (MAXss) of each lipid measure as a predictor of diabetes.

**Result:**

We found 366 (146 men and 220 women) new diabetes cases during follow-up. The risk-factor-adjusted ORs for a 1 SD increase in TG, TC/HDL-C and TG/HDL-C were 1.23, 1.27 and 1.25 in men; the corresponding risks in females were 1.36, 1.14, 1.39 respectively (all p < 0.05, except TC/HDL-C in females which was marginally significant, p = 0.07). A 1 SD increase of HDL-C only in women decreased the risk of diabetes by 25% [0.75(0.64-0.89)]. In both genders, there was no difference in the discriminatory power of different lipid measures to predict incident diabetes in the risk factor adjusted models (ROC ≈ 82%). TG cutoff values of 1.98 and 1.66 mmol/l; TG/HDL-C cutoff values of 4.7 and 3.7, in men and women, respectively, TC/HDL-C cutoff value of 5.3 in both genders and HDL-C cutoff value of 1.18 mmol/l in women yielded the MAXss for defining the incidence of diabetes.

**Conclusion:**

TC/HDL-C and TG/HDL-C showed similar performance for diabetes prediction in men population however; among women TG/HDL-C highlighted higher risk than did TC/HDL-C, although there was no difference in discriminatory power. Importantly, HDL-C had a protective effect for incident diabetes only among women.

## Background

The prevalence and incidence of type 2 diabetes (hereafter diabetes) are high in the Middle Eastern countries [1-3], which are estimated, will have the largest increases in the prevalence of diabetes by 2030 [4].The prevalence of Type 2 diabetes is reported to be over 14% in Tehran, Iran, with an estimated incidence of new cases in about 1% per year [2,3]. This could be due to changes in life style including "nutrition transition" and very low levels of physical activity that, in recent years have been the consequences of rapid urbanization and technological transition, leading to a rapid rise in risk factors of diabetes like dyslipoproteinemia [5,6].

The prevalence of dyslipoproteinemia among a Tehranian adult population was reported to be high i.e. ≥55% total cholesterol (TC) ≥5.18 mmol/l, 47% triglycerides (TG) ≥1.69 mmol/l and 30% of men and 13% of women with high density lipoprotein cholestrol (HDL-C) <0.9 mmol/l [7]. The association of high TG and/or low HDL-C with incident diabetes is well known as American Diabetes Association (ADA) recommended "screening of adults for diabetes at any age with body mass index (BMI) ≥25 kg/m^2^, HDL-C level <0.90 mmol/l and/or a TG level >2.82 mmol/l"[8]. Similarly, in Iranian adults, high TG was shown to be an independent predictor of incident diabetes [3]. Despite this, to best of our knowledge, no study has specifically assessed the diabetes risk associated with lipid ratios (i.e. TC/HDL-C and TG/HDL-C). Hence, the aim of this study was to compare head -to head the independent association between the different lipid indices with incident diabetes during ≈6 years follow up in Iranian adult men and women. Furthermore, we aimed to determine the appropriate cutoff values of these measures for incident diabetes in both genders.

## Methods

### Study population

**Figure 1:**
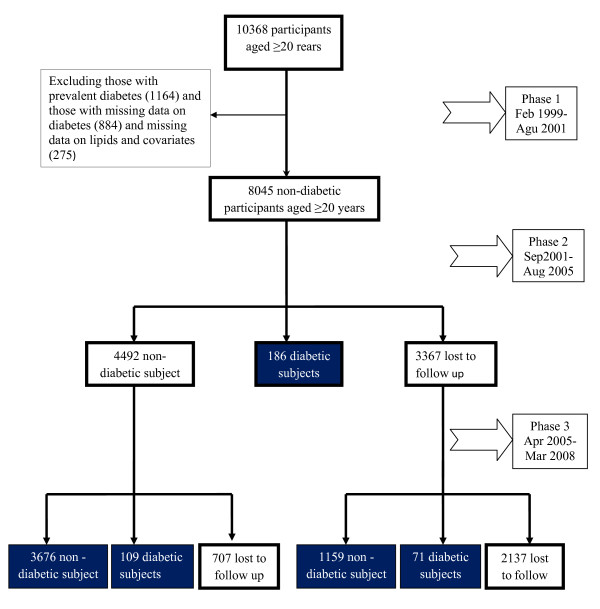
**Follow up status of the TLGS participants after the baseline examination**.

Subjects in this study were selected from among participants of the Tehran Lipid and Glucose Study (TLGS), a prospective study to determine the risk factors and outcomes for non-communicable diseases [9,10]. To summarize, 15005 people aged 3 years and over, residents of district-13 of Tehran, underwent a baseline examination in February 1999 to August 2001. After this cross-sectional phase, subjects were categorized into the cohort and intervention groups, the latter to be educated for implementation of life style modifications. 10368 participants aged 20 years and more were selected to be included into current study. After exclusion of subjects with prevalent diabetes at baseline (n = 1164), those with missing data regarding fasting and 2-hours plasma glucose (n = 884) and those with missing data for any of lipids, blood pressure measures, family history of diabetes, smoking behavior, BMI and waist circumference (WC) (n = 275), there were 8045 non-diabetic subjects which were re-assessed in the 2 consecutive phases at approximately 3-year intervals; the second phase examinations carried out between September 2001 and August 2005, and the third phase between April 2005 and March 2008 (Figure 1). Finally, 5201 subjects who developed diabetes (in phase 2 or 3) or completed the phase 3 examinations were included in this study. The ethical committee of the Research Institute for Endocrine Sciences approved this study and informed written consent was obtained from all subjects.

### Clinical and laboratory measurements

The baseline information including demographic data, medications use, family history of diabetes, past medical history of cardiovascular dieses (CVD) and smoking behavior were collected by a trained interviewer, using a pretested questionnaire. Weight was measured, while subjects were minimally clothed without shoes, using digital scales (Seca 707, Seca Corp., Hanover, MD; range 0.1-150 kg) and recorded to the nearest 100 g. Height was measured in a standing position without shoes, using tape meter with shoulders in normal alignment. BMI was calculated as weight (kg) divided by square of height (m^2^). WC was measured at umbilical level, using an unstretched tape meter, without any pressure to body surface over light clothing. Two measurements of systolic and diastolic blood pressure (SBP and DBP, respectively) were taken using a standardized mercury sphygmomanometer (calibrated by the Iranian Institute of Standards and Industrial Researches) on the right arm, after a 15 minute rest in a sitting position; mean of the two measurements was considered as subject's blood pressure. A blood sample was drawn between 7:00 and 9:00 AM from all study participants after 12-14 h overnight fasting. All the blood analyses were done at the TLGS research laboratory on the day of blood collection. Plasma glucose was measured using an enzymatic colorimetric method with glucose oxidase. The standard oral glucose tolerance test (OGTT) was performed for all participants not on glucose-lowering drugs. TC was assayed using enzymatic colorimetric method with cholesterol esterase and cholesterol oxidase. HDL-C was measured after precipitation of the apolipoprotein B-containing lipoproteins with phosphotungstic acid. TG was assayed using an enzymatic colorimetric method with glycerol phosphate oxidase. These analyses were performed using commercial kits (Pars Azmoon Inc., Tehran, Iran) and a Selectra 2 auto analyzer (Vital Scientific, Spankeren, The Netherlands). Non-HDL-C was calculated by subtracting HDL-C from TC; TC/HDL-C and TG/HDL-C were calculated by dividing TC and TG to HDL-C respectively. The intra- and inter-assay coefficients of variation (CV) were both 2.2% for glucose. For both total and HDL-Cholesterol, intra- and inter-assay CVs were 0.5 and 2% respectively. Intra- and inter-assay CVs were 0.6 and 1.6% for TG respectively [9,10].

### Definition of terms

Positive family history of diabetes was defined as having at least one parent or sibling with diabetes. History of CVD was defined any history of ischemic heart disease and/or cerebrovascular accidents. Based on ADA definitions of diabetes, those who used anti-diabetic drugs or had fasting plasma glucose (FPG) ≥7 mmol/l or 2 hours plasma glucose (2 hPG) ≥11.1 mmol/l were considered as diabetic individuals and those with 2 hPG between 7.77 and 11.1 mmol/l were defined as impaired glucose tolerance (IGT) [11]. The other diabetes risk factors were defined as follows: General obesity: BMI < 25 = normal weight, 25-29.9 = overweight and ≥30 kg/m^2 ^= obese; abdominal obesity: WC ≥90 cm in both genders, applying Iranian cutoff, at risk for CVD risk factors requiring life style changes [12]; hypertension: blood pressure ≥140/90 mmHg and/or taking of antihypertensive medication. Smoking: "current" smokers included a record of current regular or occasional smoking, "past" smokers were those who used to smoke in the past, while those who had never smoked were called "never" smokers.

### Statistics

Mean (standard deviation: SD) values for continuous and frequencies (%) for categorical variables of the baseline characteristics including lipid measures are given for participants with and without incident diabetes. Since TG and TG/HDL-C had skewed distribution they are shown as median (interquartile range). Comparison of baseline characteristics between participants with and without diabetes was done by student's t-test for continuous variables, chi-square test for categorical variables and Mann-Whitney test for skewed variables. Logistic regression analysis was used to study the associations of lipid measures with incident diabetes. Cox proportional hazards models for interval censored data, were used as alternate analyses (with Epi package in R program); the results were essentially identical with logistic models and only logistic regression results are presented.

To select covariates to be included in the multivariate logistic regression models, univariate analysis was used for each candidate covariate (age, history of CVD, family history of diabetes, intervention group, hypertension, abdominal obesity, general obesity, smoking status, IGT and lipid lowering drugs use); following this, each covariate that had a P value less than 0.2 in the univariate analysis, was selected to be included in a stepwise backward (P remove:0.1) multivariate logistic regression analysis. In men, final covariates were hypertension, general obesity, IGT and family history of diabetes; in women these were age, abdominal obesity, general obesity, IGT and family history of diabetes. Each final model included these covariates plus one of the lipid measures (TG and TG/HDL-C were added as log-transformed value). Age and multivariate odds ratios (ORs), with 95% confidence intervals (CI), were calculated for every 1 SD increase in the value of each lipid parameters. Model fitness was assessed with Hosmer-Lemeshow test; statistically significant X^2 ^statistics from this test indicate poor model fitness. The predictive ability of each model was examined with area under the receiver operating characteristic (aROC) curves and compared with "roccomp" STATA command.

To determinate appropriate cutoff values of each lipid measure for predicting diabetes, the ROC curve analysis was used with an estimation of the variable's sensitivity and specificity. In both genders, the cutoff point of each lipid variable, was assessed by the minimum value of √ (1-sensitivity)^2 ^+ (1-specificity)^2 ^[13] which representes the maximum sums of sensitivity and specificity (MAXss).

SPSS program (SPSS Inc., Chicago, IL, USA; Version 15) and STATA software (Version 10) were used for data analysis and P-values <0.05 were considered statistically significant.

### Results

The study population included 2173 men and 3028 women with mean age of 43.3 and 40.8 years, respectively. After a median follow-up of 6.4 years, 366 (146 men and 220 women) new cases of diabetes were identified.

Subjects followed, compared with those non-followed had higher rate of baseline positive family history of diabetes, general and abdominal obesity and lower rate of smoking behavior and participation in the intervention group. No significant difference was found between the two groups in baseline hypertension and IGT (data not shown).

The baseline characteristics of men and women based on incident diabetes are shown in Table 1.

**Table 1:** Baseline characteristics of participants with and without incident diabetes in men and women

	Men			Women		
		
	Incident diabetes (146)	No diabetes (2027)	P Value	Incident diabetes (220)	No diabetes (2808)	P Value
Age (year)	49.1 ± 13.5	42.8 ± 14.0	<0.001	46.6 ± 11.7	40.3 ± 12.5	<0.001
Family history of diabetes (%)	39.7	24.0	<0.001	42.3	26.4	<0.001
Intervention (%)	39.7	35.4	0.3	35.0	36.2	0.7
History of CVD (%)	6.8	3.9	0.09	5.9	2.3	0.001
Smoking (%)			0.9			0.5
Never	62.3	60.5		94.5	96.2	
Past	13.7	13.7		1.8	1.2	
Current	25.0	25.8		3.6	2.5	
Hypertension (%)	37.7	17.5	<0.001	36.8	18.0	<0.001
Obesity (%)			<0.001			<0.001
Normal weight	21.2	43.7		11.4	31.7	
Overweight	47.3	43.4		34.5	40.9	
Obese	31.5	13.0		54.1	27.4	
Abdominal obesity (%)	67.8	45.1	<0.001	72.3	41.0	<0.001
Lipid-lowering drug use (%)	1.5	2.1	0.6	6.4	2.6	0.001
Impaired glucose tolerance (%)	54.1	9.5	<0.001	55.9	11.4	<0.001
Triglycerides (mmol/l)	2.1 (1.5-3.0)	1.7(1.8-2.4)	<0.001	2.1 (1.4-3.0)	1.4(1.0-2.1)	<0.001
Total cholesterol (mmol/l)	5.6 ± 1.2	5.3 ± 1.1	<0.001	5.6 ± 1.3	5.4 ± 1.2	<0.001
HDL-C (mmol/l)	0.9 ± 0.3	1.0 ± 0.2	0.2	1.1 ± 0.2	1.7 ± 0.3	<0.001
Non-HDL-C (mmol/l)	4.7 ± 1.2	4.3 ± 1.1	<0.001	4.8 ± 1.3	4.2 ± 1.2	<0.001
TC/HDL-C	6.2 ± 2.2	5.6 ± 1.6	<0.001	5.7 ± 1.8	4.9 ± 1.6	<0.001
TG/HDL-C	5.1(3.4-8.2)	4.0(2.5-6.3)	<0.001	4.5(2.8-7.0)	2.7 (1.8 -4.5)	<0.001

CVD: Cardiovascular disease, HDL-C: High density lipoprotein cholesterol, Mean ± SD are shown for continuous variables and P value is calculated with t-test; % is shown for categorical variables with P value according to chi-square; TG and TG/HDL are shown as median (interquartile range) and P value according to Mann-Whitney test. For categorical variables with more than 2 values, the first category was considered as the reference and the p-value is for linear trend in risk. Obesity: BMI < 25 (normal), 25-29.9 (overweight) and ≥30 kg/m2 (obese), Abdominal obesity: waist circumference ≥90 cm in both sexes; Hypertension: Blood pressure ≥140/90 mmHg and/or taking of antihypertensive medication; IGT: 2-hours plasma glucose level between 7.77-11.1 mmol/l.

The men who developed diabetes were older compared to those without diabetes and had higher prevalence of family history of diabetes, hypertension, general and abdominal obesity and IGT. All baseline lipid measures levels, except for HDL-C, were higher in men with incident diabetes compared to those without it. Women with incident diabetes were older than those without this condition. Rates of positive family history of diabetes, history of CVD, hypertension, general and abdominal obesity, lipid-lowering drug use and IGT were higher in women who developed diabetes compared those did not. All lipids values were higher in women with incident than in women without incident diabetes, except HDL-C level which was higher in subjects without incident diabetes.

The ORs of a 1 SD change in each lipid marker or index, in men and women, are presented in Table 2. In men, in the age adjusted model, there was positive association between TC, TG, non-HDL-C, TC/HDL-C and TG/HDL-C with incident diabetes; but in the fullly adjusted model, only, TG, TC/HDL-C and TG/HDL-C were independent predictors of diabetes and a 1 SD increase in each of these lipids increased risk of diabetes by 23%, 27% and 25%, respectively. In women, a 1 SD change of all lipid measures, except for TC, resulted in a significant risk of incident diabetes in age adjusted model. However, after further adjustment, HDL-C, TG and TG/HDL-C remained as independent predictors of diabetes; a 0.3 mmol/L increase in HDL-C decrease the risk of diabetes by 25% and a 1 SD increase in log-transformed TG and TG/HDL-C, resulted 36% and 39% increased risk of incident diabetes in multivariate adjusted model in women. Additionally, in this group, a 1.7 increase in TC/HDL-C increased the risk of diabetes ≈14% in full adjusted analysis which was marginally significant (P = 0.07).

**Table 2:** Odds ratios of lipid measures for incident diabetes mellitus

		Age adjusted model		multivariate adjusted model			
			
	SD mmol/l	Odds ratio (95%CI)	P value	Odds ratio (95%CI)	P value	**H-L X**^**2 **^**(P value)**	aROC (95%CI)
**men**							
TC	1.1	1.28(1.09-1.52)	0.002	1.11(0.93-1.33)	0.2	5.65(0.7)	0.81
HDL-C	0.2	0.86(0.72-1.02)	0.1	0.91(0.75-1.09)	0.3	5.05(0.7)	0.81
TG	0.6*	1.62(1.37-1.92)	<0.001	1.23(1.02-1.49)	0.03	5.53(0.7)	0.82
Non-HDL-C	1.1	1.34(1.13-1.58)	<0.001	1.14(0.95-1.37)	0.15	6.59(0.6)	0.81
TC/HDL-C	1.7	1.44(1.22-1.69)	<0.001	1.27(1.06-1.51)	0.007	2.97(0.9)	0.82
TG/HDL-C	0.7*	1.44(1.25-1.65)	<0.001	1.25(1.03-1.52)	0.02	6.11(0.6)	0.82
**women**							
TC	1.2	1.15(0.98-1.32)	0.1	0.94(0.80-1.12)	0.52	5.52 (0.7)	0.81
HDL-C	0.3	0.66(0.56-0.76)	<0.001	0.75(0.64-0.89)	0.001	3.67(0.9)	0.82
TG	0.5*	1.74(1.50-2.01)	<0.001	1.36(1.13-1.58)	0.001	7.94(0.4)	0.82
Non-HDL-C	1.2	1.26(1.09-1.45)	0.003	1.01(0.86-1.19)	0.86	4.34(0.8)	0.81
TC/HDL-C	1.7	1.37(1.21-1.55)	<0.001	1.14(0.99-1.31)	0.07	5.66(0.7)	0.81
TG/HDL-C	0.7*	1.78(1.53-2.06)	<0.001	1.39(1.17-1.64)	<0.001	7.47(0.5)	0.82

TC: total cholesterol, HDL-C: high density lipoprotein cholesterol, TG: triglycerides, SD: standard deviation, CI: confidence interval. aROC: area under the receiver operating characteristic of each model. Odd ratios indicate the increase risk for a 1 SD increase of each lipid parameter Multivariate models were adjusted for hypertension, family history of diabetes, general obesity and impaired glucose tolerance in men and for age, family history of diabetes, general obesity, abdominal obesity and impaired glucose tolerance in women. H-L X^2 ^indicates Hosmer and Lemeshow goodness of fit test. * SD of log-transformed.

The Hosmer-Lemeshow test did not reject the goodness of fit for the models (all P > 0.4). Predictive ability (as assessed by aROC) of these models were statistically equal (all P value > 0.05) in each gender.

Table 3 presents the cutoff points of lipid measures for prediction of diabetes with their corresponding specificity and sensitivity in men and women. Cutoff points of TG, TC/HDL-C and TG/HDL-C to predict diabetes in men and women were 1.98 and 1.66 mmol/l, 5.3 and 5.3, 4.7 and 3.7, respectively. Also the corresponding cutoff value of HDL-C in women was 1.18 mmol/l.

**Table 3:** Receiver operator curve characteristics for significant lipid measures in predicting diabetes and cutoff points for maximum sum of sensitivity and specificity in men and women

	Men				Women			
		
	aROC (95% CI)	Cut point	Sensitivity (%)	Specificity (%)	aROC (95% CI)	Cut point	Sensitivity (%)	Specificity (%)
TG	0.62(0.57-0.66)	1.98	0.57	0.61	0.67 (0.65-0.72)	1.66	0.67	0.60
HDL-C					0.60 (0.57-0.64)	1.18	0.49	0.36
TC/HDL-C	0.59(0.53-0.63)	5.3	0.65	0.48	0.65 (0.61-0.68)	5.3	0.57	0.66
TG/HDL-C	0.60(0.56-0.65)	4.7	0.57	0.59	0.69 (0.65-0.72)	3.7	0.66	0.64

aROC: area under the receiver operating characteristic; TC: total cholesterol, HDL-C: high density lipoprotein cholesterol, TG: triglycerides. Cut points are presented as mmol/l

We also used a method for defining the cutoff points of lipid measures to facilitate the identification of 70% of future cases of diabetes (that is, a sensitivity of ≈70%) (Table 4). The results, as expected, produced lower cutoff points of TG, TG/HDL-C and TC/HDL-C and higher cut point of HDL-C in predicting diabetes.

**Table 4:** Receiver operator curve characteristics for significant lipid measures in predicting diabetes and cutoff points for sensitivity ≈70% in men and women

	Men		Women	
		
	Cut point	Specificity (%)	Cut point	Specificity (%)
TG	1.61	45	1.54	55
HDL-C			1.19	50
TC/HDL-C	5.1	43	4.77	53
TG/HDL-C	3.66	44	3.4	59

TC: total cholesterol, HDL-C: high density lipoprotein cholesterol, TG: triglycerides. Cut points are presented as mmol/l

## Discussion

In a population with high prevalence and incidence of diabetes, our findings highlighted TG, TG/HDL-C and TC/HDL-C as independent predictors of incident diabetes, among men, during ≈6 years follow up with the ORs for a 1 SD increase in these lipid measures ranging from 23 to 27%. Furthermore, among women, TG, TC/HDL-C and TG/HDL-C were independent predictors with the ORs ranging from 14% for TC/HDL-C (marginally significant) to ≈40% for TG/HDL-C. HDL-C only among women was an independent protective lipid measure for incident diabetes. Our study showed that in this study population, the TG cutoff values of 1.98 and 1.66 mmol/l; TG/HDL-C cutoff values of 4.7 and 3.7, in men and women, respectively, TC/HDL-C cutoff value of 5.3 in both genders and HDL-C cutoff value of 1.18 mmol/l in women yielded the MAXss for defining the incidence of diabetes.

In the current study, serum TG was a strong predictor of incident diabetes in both genders; independent of the other risk factors. The association of fasting TG with incident diabetes has been documented previously [14,15]. However, this was reported largely when TG levels were pooled with additional risk factors for diabetes or cardiovascular disease [16]. However, Tirosh et al highlighted that two measurements of fasting triglyceride levels with 5 years interval can predict incident diabetes in healthy young men independent of traditional risk factors [16]. Furthermore, the cut off points of TG in men (≈1.92 mmol/l) and women (≈1.69 mmol/l) to predict incident diabetes in this study of an Iranian population were clearly lower than those recommended by ADA for screening diabetes [8], nevertheless, these values were closer to the TG criteria of metabolic syndrome (i.e. 1.69 mmol/l) [17].

Low HDL-C is known to be an important predictor for development of diabetes [18], and certain agents known to raise HDL-C improve glucose metabolism and prevent diabetes [19]. In the current study, serum HDL-C was a strong independent negative risk factor of diabetes in women. An increment of 0.3 mmol/l in HDL-C was associated with a 25 percent lower risk of incident diabetes among women but did not predict diabetes in men even in age adjusted analysis. Similarly, in Finnmark Study, a significant predictive relevance of HDL-C was shown only in women [20]. In contrast to our results, some studies showed that HDL-C had a significant relationship with incident diabetes in men but not in women [21,22]; Also there were some evidences demonstrating HDL-C was inversely associated with diabetes in both genders [23].

Despite the controversy, accumulating evidence indicates that both low HDL-C and hypertriglyceridemia which previously were known as early manifestations of insulin resistance (IR) and later diabetes, can actively add to β cell failure and thus to the appearance of diabetes [24].

Although several studies demonstrated that lipid ratios, such as TC/HDL, could be better predictors of CVD than any single lipid measures [25], there is lack of data regarding lipid ratios for predicting diabetes. Kimm et al showed that lipid ratios including TC/HDL and TG/HDL were associated with IR and might be used as integrated lipid values [26]. Jespersen et al in a cross sectional study among normal and overweight non-diabetic individuals showed that those with high value of TC/HDL-C were more resistant to insulin-stimulated glucose disposal, and to had higher blood pressure, increased TG concentrations, and hyperinsulinemia [27], rendering these individuals at increased risk of coronary heart disease and possibly diabetes for reasons unrelated to cholesterol metabolism. Among obese individuals, however, Brehm et al showed that these relationships do not exist [28]. In the current study, the multivariate OR associated with a 1 SD increase in TC/HDL-C was approximately 1.27 and 1.14 in men and women, respectively; yielding a similar cutoff point of 5.3 to predict incident diabetes.

It was shown in the previous studies that TG/HDL-C is as close as fasting plasma insulin concentration with IR and could be used as an indicator of IR in clinical setting [29,30]; as TG/HDL > 3.5 is a strong indicator of the presence of IR. This ratio correlated well with the insulin suppression test and was effective as the ATP III MS criteria in identifying IR individuals [29,30]. Recently we showed that prevalence of metabolic syndrome in Iranian men with TG/HDL-C level of 2.8-4.4 is five-fold higher than those with TG/HDL-C < 2.8 [31]. Furthermore, TG/HDL-C ≥ 3.5 was an independent predictor of incident diabetes and along with SBP, family history of diabetes, waist-to-height ratio and fasting plasma glucose levels ≥5 mmol/l showed aROC of 0.83 (95% CI 0.80-0.86) to discriminate subjects at substantial risk for diabetes among Iranian population [32]. In our data analyses, a 1 SD increase in TG/HDL-C yielded higher risk for incident diabetes in women than in men (39% vs. 25%) and also a lower cutoff point in women showed more sensitivity and specificity than in men. McLaughlin et al, however, did not identify any interaction between sex and the ability of the TG/HDL-C ratio to predict IR in 127 men and 131 women [29]. Generally, considering effect sizes (ORs with CI) and model discrimination (as assessed by aROC), TC/HDL-C and TG/HDL-C showed similar performance for diabetes prediction in men, however; among women TG/HDL-C highlighted higher risk than did TC/HDL-C, although there was no difference in discriminatory power. On the other hand, TC/HDL-C besides being as inexpensive and simple to measure as the TG/HDL-C has the additional benefit of its biologic inconsistency being much lower and fasting state is not needed [33].

In our data analysis, we used ROC analysis to calculate the cutoff points of lipid variables based on equally weighted sensitivity and specificity [13], hence these cutoff points had low sensitivity for predicting diabetes in clinical practice. Additionally, these points may not be the optimal cutoff in the clinic because sensitivity versus specificity should be weighted against many other factors such as seriousness of the illness, how invasive or feasible it might be, the test used for first evaluation and finally how often the test should be performed [34]. Furthermore, to cover a reasonable proportion of the population at risk, we calculated the cutoff point of each lipid measure with sensitivity at the level of at least 70% (not to miss more than 30% cases of incident diabetes) and as expected, produced lower cutoff points of lipid parameters with lower specificities.

There are potential limitations regarding the interpretation of our results. First, we measured TC, HDL-C and TG only once at the baseline per subject, thus the potential bias resulting from regression dilution of TG and HDL-C could not be ignored [35], additionally, given the significant intraindividual variation in fasting TG level, it might be possible that a proportion of participants who had a high concentration of TG are falsely classified as low, and vice versa; hence, attenuating the true relative risk of the lipoprotein. Second some misclassification of diabetes status may have occurred due to defining new diagnosed diabetes with a single OGTT at baseline or follow up. However, the extent of misclassification would have to be extensive to change the results of this study [36]. Third, we did not evaluate low-density lipoprotein cholesterol because it was calculated, rather than measured, in TLGS (i.e. requiring exclusion of participants with TG ≥ 4.52 mmol/l), although it has not been reported to have a strong correlation with IR in the literature [37]. Furthermore, we did not evaluate apolipoproteins because they have not been as routinely used as TC, TG and HDL-C as predictors of IR and incident diabetes.

As strength, to our best knowledge, this study was the first prospective and population-based study, which examined different lipid measures simultaneously for prediction of diabetes in both genders.

## Conclusions

TC/HDL-C and TG/HDL-C showed similar performance for diabetes prediction in men population; however, among women TG/HDL-C highlighted higher risk than did TC/HDL-C, although there was no difference in discriminatory power. Importantly, HDL-C had a protective effect for incident diabetes only among women. Hence, although we cannot infer causality from our observational study, it is worth speculating that lowering TG/HDL-C and TC/HDL-C levels in both genders either pharmacologically or through lifestyle modification, may constitute a viable means to attenuate diabetes risk in Iranian population.

## Competing interests

The authors declare that they have no competing interests.

## Authors' contributions

FH participated in the conception and design of the study; interpretation of data and drafting the manuscript. MH participated in statistical analysis and interpretation of data and drafting the manuscript. MT revised the manuscript for important intellectual content. PS participated in the statistical analyses. NS revised the manuscript for important intellectual content. FA participated in its design and coordination. All authors read and approved the final manuscript.
